# Supplementary material for: The Patterns of Codon Usage between Chordates and Arthropods are Different but Co-evolving with Mutational Biases
Source: Mol Biol Evol. 2024 Apr 26;41(5):msae080. doi: 10.1093/molbev/msae080 (PMC11108087; doi:10.1093/molbev/msae080)
Supplement: msae080_Supplementary_Data [file msae080_supplementary_data.zip › MBE-23-0357.R2_supp-figures.pdf]

Supplementary Figures

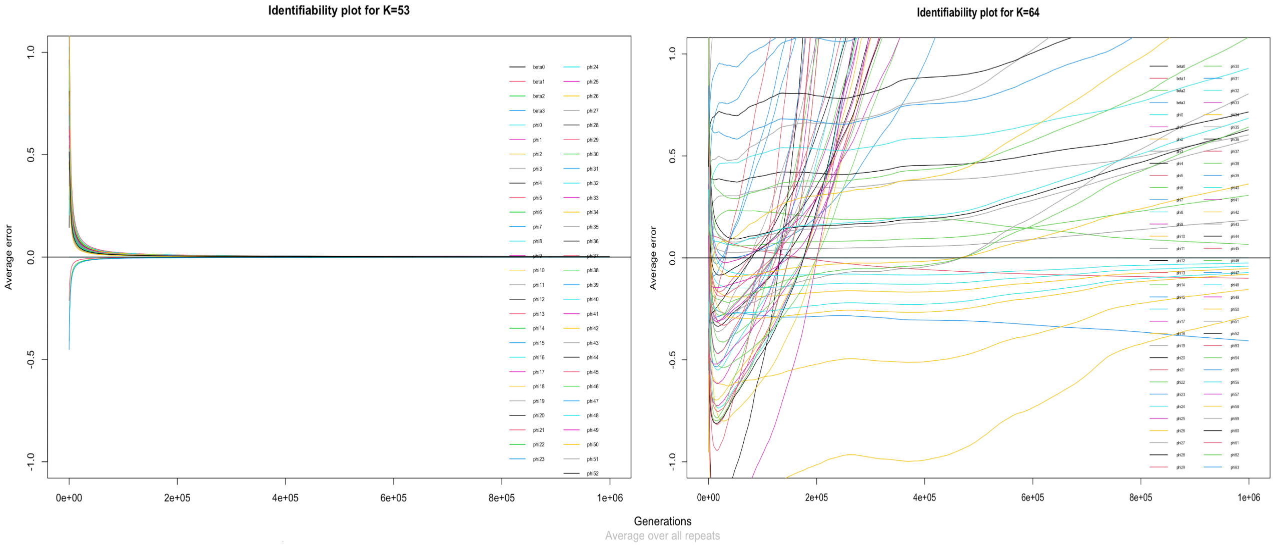

Figure S1: Cumulative sum of errors of each parameter estimate over the MCMC generations. The plot on the left represents the cumulative errors of simulated mutational bias parameters and 53 simulated fitness coefficients ($K = 53$; identifiability threshold). After 53 coefficients the model becomes unidentifiable, like the plot on the right with $K = 64$ fitness coefficients (one codon coefficient per codon). Both plots show the cumulative errors averaged over 8 repeats for 1 million MCMC generations.

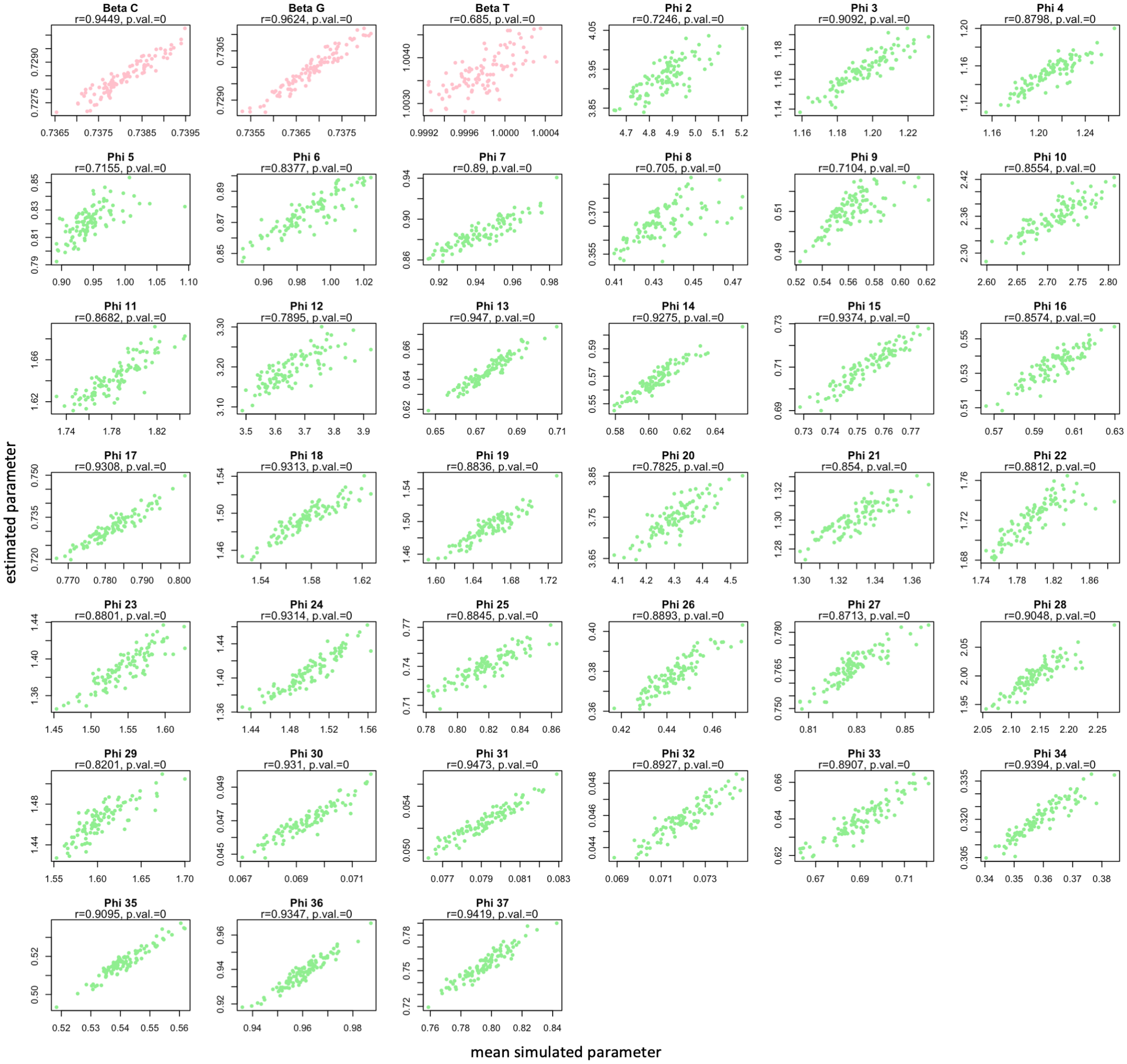

Figure S2: Relationship between mean simulated values and estimations in *Drosophila melanogaster*. Comparison of average parameter values simulated across 10,000 fruit fly genes and those estimated by DECUB. Spearman's ρ correlation coefficient is provided alongside its corresponding p-value. Mutational biases are denoted by pink dots, and codon fitnesses are represented by green dots.

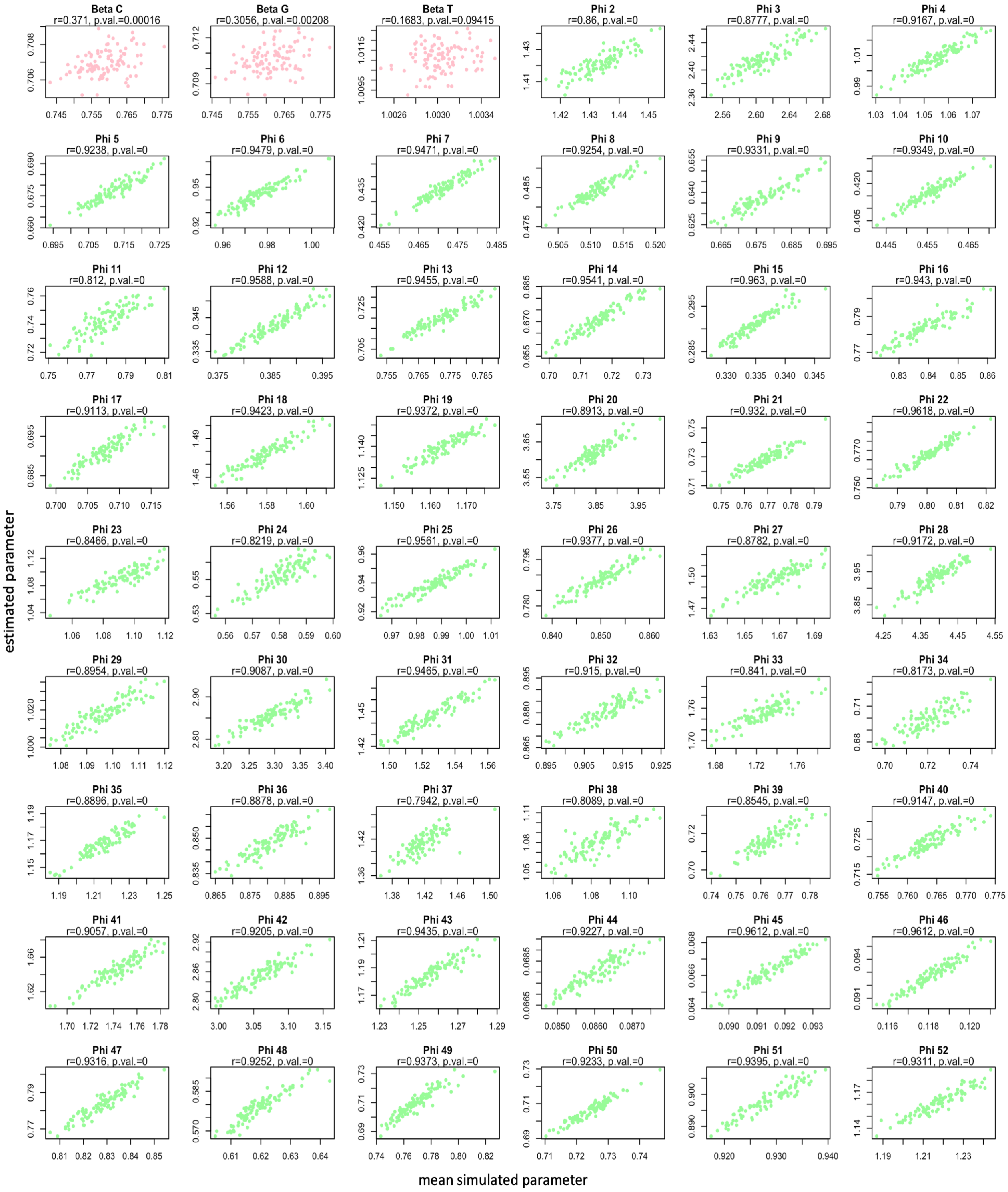

Figure S3: Relationship between mean simulated values and estimations in *Homo sapiens*. Comparison of average parameter values simulated across 20,000 human genes and those estimated by DECUB. Spearman's ρ correlation coefficient is provided alongside its corresponding p-value. Mutational biases are denoted by pink dots, and codon fitnesses are represented by green dots.

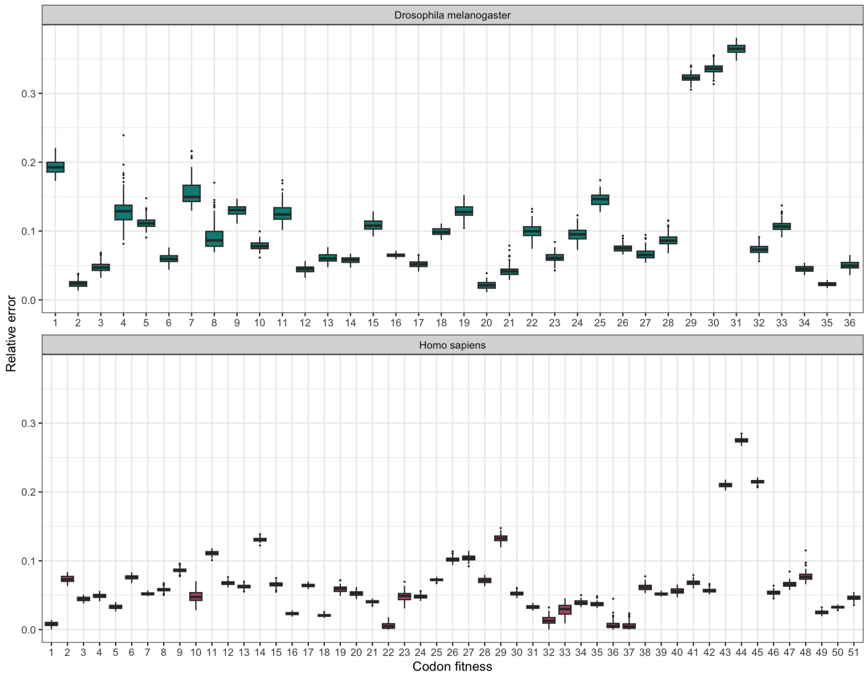

Figure S4: Relative error between mean simulated codon fitnesses and estimated ones in *Drosophila melanogaster* and *Homo sapiens*. Relative error between estimated and average simulated codon fitnesses across 10,000 genes in fruit flies and 20,000 genes in humans.

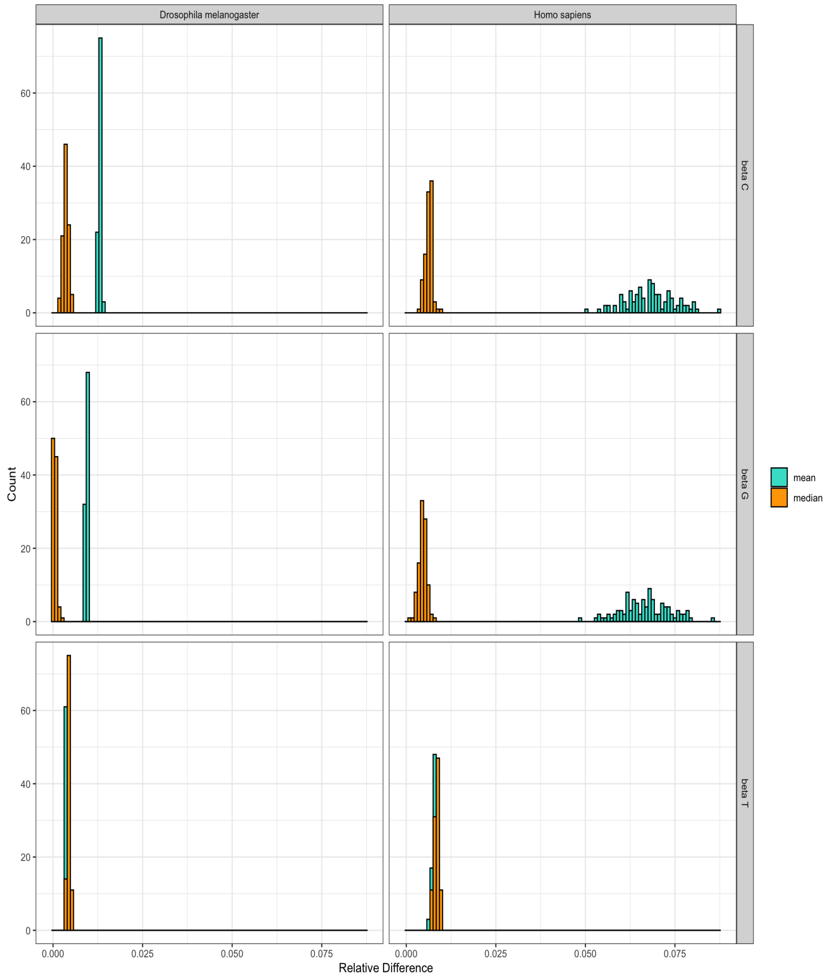

Figure S5: Comparison of relative errors between mean and median simulated mutational biases and their respective estimated values in *Drosophila melanogaster* and *Homo sapiens*. Relative error between estimated and mean simulated mutational biases (shown in blue) and the median (shown in orange) across 10,000 genes in fruit flies and 20,000 genes in humans.

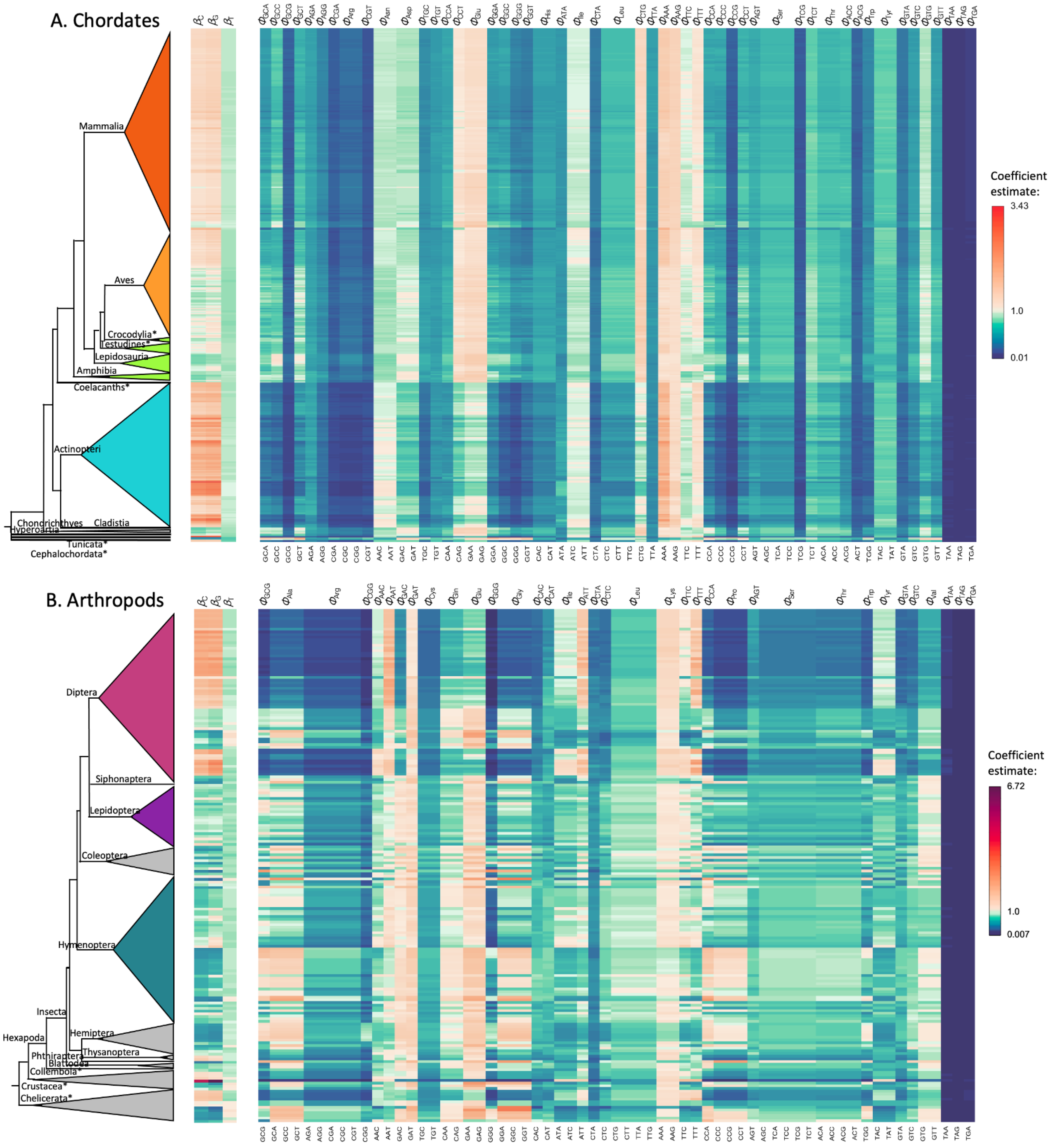

Figure S6: Image plots of parameter estimates after the final mapping for chordates and arthropods. Parameter estimate for each mutational bias coefficient β and fitness coefficient Φ (top of image plots). The codons corresponding to the fitness coefficients are shown at the bottom of the image plots and the colours represent the magnitude of the estimate with white being equal to the reference, blue disfavoured and red shows the favoured estimates. Finally, on the left, the corresponding phylogeny of the species is shown for (A) chordates and (B) arthropods.

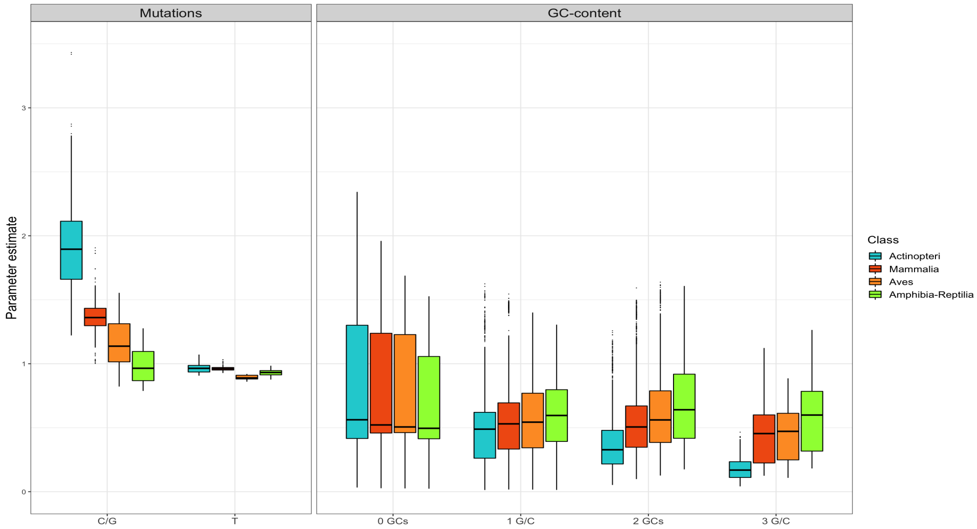

Figure S7: Mutational biases and fitness coefficient estimates in chordates. Grouped mutational estimates for C/G and A/T mutational biases and fitness coefficient estimates grouped according to the GC–content of each respective codon. Only the codons with a specific fitness coefficient were considered (excluding fitness coefficients of grouped codons encoding the same amino acid).

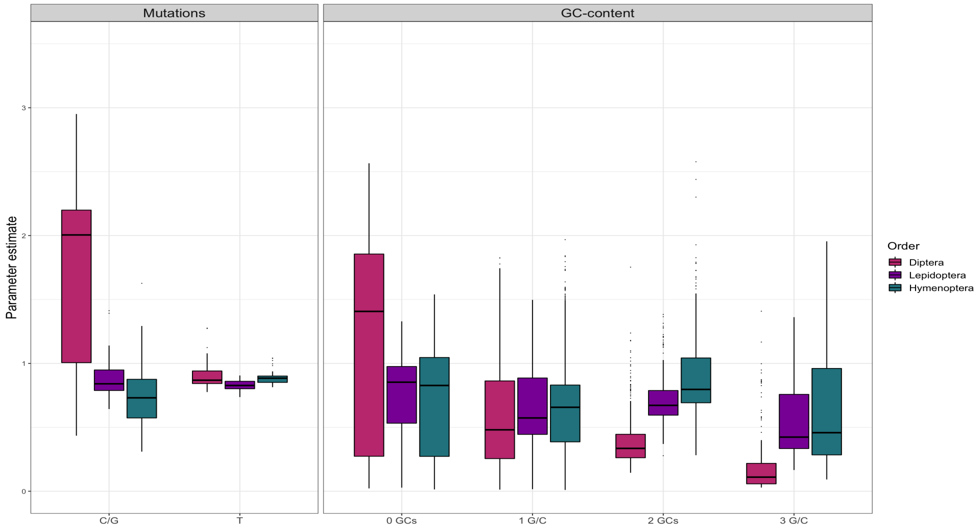

Figure S8: Mutational biases and fitness coefficient estimates in arthropods. Grouped mutational estimates for C/G and A/T mutational biases and fitness coefficient estimates grouped according to the GC–content of each respective codon. Only the codons with a specific fitness coefficient were considered (excluding fitness coefficients of grouped codons encoding the same amino acid).

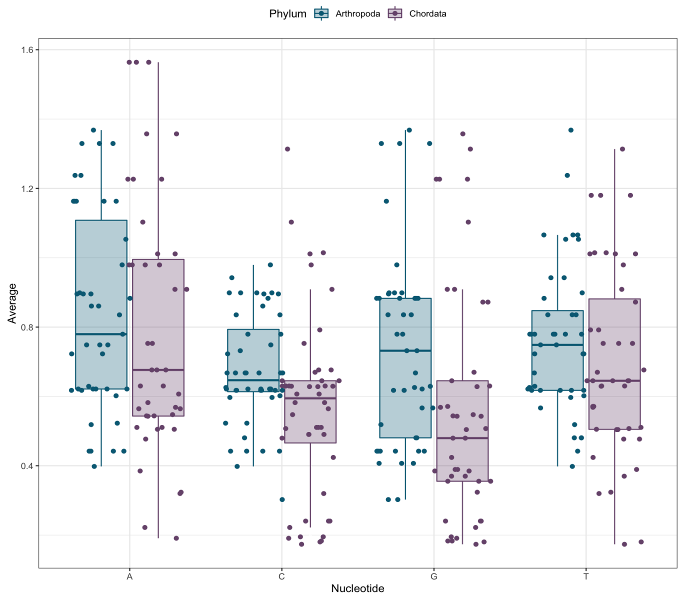

Figure S9: Distribution of fitness coefficients per nucleotide in arthropods and chordates. Boxplots showing no association between the distribution of fitness coefficients and their nucleotide composition. This plot helps demonstrate that fitness coefficients are not compensating for the mutation parameters. This comparison was validated using a Wilcoxon test with FDR correction. From the 12 comparisons, only the distribution of fitness coefficients of nucleotides A and G in chordates show significant differences (p–value=0.0056).

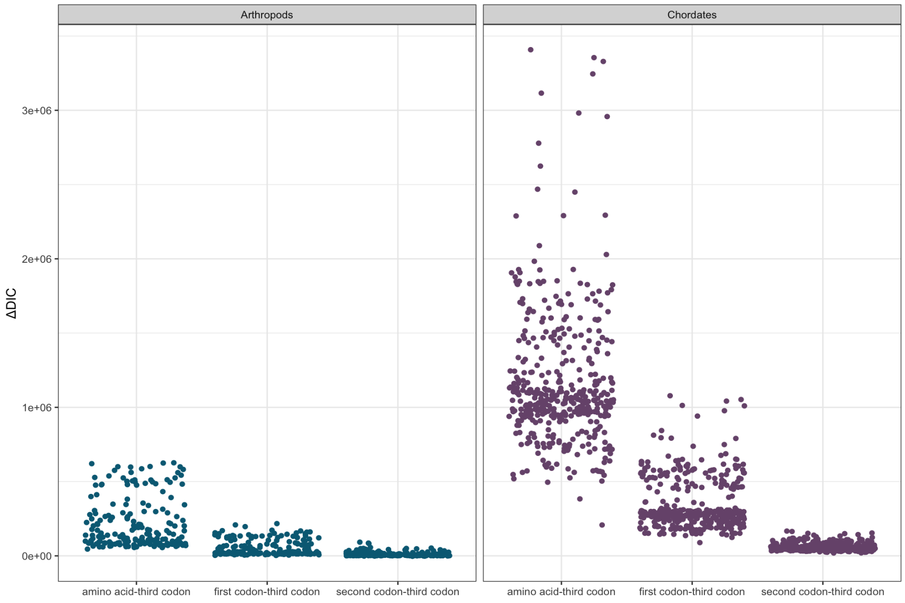

Figure S10: ΔDIC values between previous mapping and the last codon mapping. Comparisons between the previous mappings with the optimal one (last codon mapping, smallest DIC value). In both arthropods and chordates, the last one was preferred with ΔDIC values > 10, the threshold for accepting the optimal model.
