# Supplementary material for: The Patterns of Codon Usage between Chordates and Arthropods are Different but Co-evolving with Mutational Biases
Source: Mol Biol Evol. 2024 Apr 26;41(5):msae080. doi: 10.1093/molbev/msae080 (PMC11108087; doi:10.1093/molbev/msae080)
Supplement: msae080_Supplementary_Data [file msae080_supplementary_data.zip › MBE-23-0357.R2_supp-tables-S3-4.pdf]

Supplementary Tables

Table S3: Species information. Number of overall species as well as per group tested (top table), and number of species, average number of genes and sites for chordates and artrhopods (bottom table).

Phylum	Taxon		# of species
**Chordata**	Total		415
	Mammalia	Mammals	163
	Aves	Birds	88
	Reptilia & Amphibia	Reptiles & Amphibians	30
	Actinopteri	Fish	117
**Arthropoda**	Total		191
	Diptera	Flies & Mosquitoes	64
	Lepidoptera	Butterflies & Moths	23
	Hymenoptera	Ants, Bees & Wasps	55

Phylum	# of species	Average # of genes	Average # of sites
**Chordata**	415	20,837.3	11,792,024
**Arthropoda**	191	14,171.1	7,491,224

Note: Reptilia includes the classes including Crocodylia, Testudines, and Lepidosauria.

Table S4: Statistical measurements between CpG (and TpG) sites and their respective GC-content. Values for the one-sample Wilcoxon signed-rank test Statistic and p-value is shown after FDR correction for the groups analysed.

**Chordata**					
		**CpG <3 GCs**		**TpG >1 GC**	
Taxon		*Statistic*	*p-value*	*Statistic*	*p-value*
Overall		538226	<2.2e-16*	4709110	<2.2e-16*
Mammalia	Mammals	1050	<2.2e-16*	792163	1.512e-14*
Aves	Birds	52	<2.2e-16*	239470	2.12e-12*
Amphibia+Reptilia	Amphibians+Reptiles	33	<2.2e-16*	37049	1.373e-07*
Actinopteri	Fish	53935	<2.2e-16*	355859	0.08656

**Arthropoda**					
		**CpG <3 GCs**		**TpG >1 GC**	
Taxon		*Statistic*	*p-value*	*Statistic*	*p-value*
Overall		25552	1	139948	0.9997
Diptera	Flies+Mosquitos	6065	1	23892	1
Lepidoptera	Butterflied+Moths	675	1	3725	1
Hymenoptera	Ants+Bees+Wasps	4152	1	23161	1
