# Supplementary material for: The Patterns of Codon Usage between Chordates and Arthropods are Different but Co-evolving with Mutational Biases
Source: Mol Biol Evol. 2024 Apr 26;41(5):msae080. doi: 10.1093/molbev/msae080 (PMC11108087; doi:10.1093/molbev/msae080)
Supplement: msae080_Supplementary_Data [file msae080_supplementary_data.zip › MBE-23-0357.R2_supp-text.pdf]

Supplementary material

Supplementary Text

S1. The model

Our model is modelled as a continuous–time Markov process based on Polymorphism–aware Phylogenetic Models, and it includes monomorphic and polymorphic states. Every polymorphic pair is biallelic and differs at a single position, as mutation rates are expected to be much lower than genetic drift in eukaryotes. Mutations are reversible and biased, as the mutation rate from codon I to J (μ_{IJ}) is not necessarily equal to the opposite mutation rate (μ_{JI}). They are assumed to happen only to escape a monomorphic state (boundary sites) and are modelled similarly to the general time–reversible (GTR) substitution model, where the mutation rates are the product of the stationary frequencies (π) and the exchangeability rates (ρ) between the four nucleotides. Genetic drift is incorporated according to the Moran Model and GC–bias as a selection coefficient favouring GC–alleles to capture the effects of GC–biased gene conversion (gBGC). Finally, selection is modelled as a relative fitness coefficient (ϕ).

If we assume two codons $I = i_1 i_2 i_3$ and $J = j_1 j_2 j_3$ differ at a singe position c, with $i_k = j_k \forall k \neq c$, then the transitional matrix Q is:

$$Q = q_{\substack{\{nI,(N-n)J\},\\ \{mI,(N-m)J\}}} = \begin{cases} \mu_{IJ} = \rho_{i_c j_c} \pi_J = \rho_{i_c j_c} \pi_{j_1} \pi_{j_2} \pi_{j_3}, & \text{if } n = N \text{ and } m = N-1 \\ \mu_{JI} = \rho_{j_c I_c} \pi_I = \rho_{j_c I_c} \pi_{i_1} \pi_{i_2} \pi_{i_3}, & \text{if } n = 0 \text{ and } m = 1 \\ \frac{n(N-n)}{N[n\gamma^{\mathbf{1}_{G,C}(i_c)}\phi_I + (N-n)\gamma^{\mathbf{1}_{G,C}(j_c)}\phi_J]} \gamma^{\mathbf{1}_{G,C}(j_c)} \phi_J, & \text{if } 1 \leq n \leq N-1 \text{ and } m = n-1 \\ \frac{n(N-n)}{N[n\gamma^{\mathbf{1}_{G,C}(i_c)}\phi_I + (N-n)\gamma^{\mathbf{1}_{G,C}(j_c)}\phi_J]} \gamma^{\mathbf{1}_{G,C}(i_c)} \phi_I, & \text{if } 1 \leq n \leq N-1 \text{ and } m = n+1 \\ 0, & \text{otherwise} \end{cases} \tag{1}$$

where n and $N - n$ describe the absolute frequencies of the two codons in the initial population of size N, m and $N - m$ are the derived frequencies, π_I is the nucleotide frequencies for codon I ($\pi_I = \pi_{i_1} \pi_{i_2} \pi_{i_3}$) and ϕ_I is the fitness coefficient of codon I (similarly for codon J). Finally, $\mathbf{1}_{G,C}(i)$ is the indicator function of nucleotide i, which guarantees that the γ parameter contributes to a given codon frequency only if it has a GC nucleotide, thus modelling for gBGC.

S2. Stationary distribution

The global balance equation characterises the stationary distribution of a Markov chain, and states that the transition rates in and out of state s_i are equal.

$$\psi_{s_i} \sum_{\forall s_i \neq s_j} q_{s_i,s_j} = \sum_{\forall s_i \neq s_j} \psi_{s_j} q_{s_j,s_i} \ , \tag{2}$$

where $\psi_{s_i} q_{s_i,s_j}$ represents the probability flux from state s_i to state s_j. So the left–hand side represents the total flow from out of state s_i into states other than s_i, while the right–hand side represents the total flow out of all states $s_j \neq s_i$ into state s_i.

In a continuous time Markov chain with a transition rate matrix $Q = q_{s_i,s_j}$, where $q_{s_i,s_i} = -q_{s_i}$ and $\psi Q = 0$, the sum of all stationary distributions is equal to 1, $\sum \psi_{s_i} = 1$. When the model is reversible, the global balance equation can be simplified to the detailed balance equations for every pair of states s_i and s_j:

$$\psi_{s_i} q_{s_i,s_j} = \psi_{s_j} q_{s_j,s_i} \tag{3}$$

To calculate the stationary distributions, we will use the rate matrix defined in eq. (1). Assuming we have two codons I and J in a population of N individuals, we have all frequency shifts defined as $q_{\{nI,(N-n)J\},\{mI,(N-m)J\}}$. Initially, as a first step assuming we start from a population of fixed for the codon I individuals ($\{NI\}$), going from the monomorphic state to the first polymorphic state ($\{(N-1)I, J\}$), based on eq. (3), we get:

$$\begin{aligned} &\psi_{\{NI\}} q_{\{NI\},\{(N-1)I,J\}} = \psi_{\{(N-1)I,J\}} q_{\{(N-1)I,J\},\{NI\}} \\ &\Leftrightarrow \psi_{\{NI\}} N \rho_{i_c j_c} \pi_J = \psi_{\{(N-1)I,J\}} \frac{(N-1)}{N[(N-1)\gamma^{\mathbf{1}_{G,C}(i_c)}\phi_I + \gamma^{\mathbf{1}_{G,C}(j_c)}\phi_J]} \gamma^{\mathbf{1}_{G,C}(i_c)}\phi_I \end{aligned} \tag{4}$$

Similarly, the same will hold going from $\{(N-1)I, J\}$ to $\{(N-2)I, 2J\}$ and opposite.

$$\begin{aligned} &\psi_{\{(N-1)I,J\}} q_{\{(N-1)I,J\},\{(N-2)I,2J\}} = \psi_{\{(N-2)I,2J\}} q_{\{(N-2)I,2J\},\{(N-1)I,J\}} \\ &\Leftrightarrow \psi_{\{(N-1)I,J\}} \frac{(N-1)}{N[(N-1)\gamma^{\mathbf{1}_{G,C}(i_c)}\phi_I + \gamma^{\mathbf{1}_{G,C}(j_c)}\phi_J]} \gamma^{\mathbf{1}_{G,C}(j_c)}\phi_J = \\ &\quad \psi_{\{(N-2)I,2J\}} \frac{2(N-2)}{N[(N-2)\gamma^{\mathbf{1}_{G,C}(i_c)}\phi_I + 2\gamma^{\mathbf{1}_{G,C}(j_c)}\phi_J]} \gamma^{\mathbf{1}_{G,C}(i_c)}\phi_I \\ &\Leftrightarrow \psi_{\{(N-1)I,J\}} \frac{(N-1)}{N[(N-1)\gamma^{\mathbf{1}_{G,C}(i_c)}\phi_I + \gamma^{\mathbf{1}_{G,C}(j_c)}\phi_J]} = \\ &\quad \psi_{\{(N-2)I,2J\}} \frac{2(N-2)}{N[(N-2)\gamma^{\mathbf{1}_{G,C}(i_c)}\phi_I + 2\gamma^{\mathbf{1}_{G,C}(j_c)}\phi_J]} \frac{\gamma^{\mathbf{1}_{G,C}(i_c)}}{\gamma^{\mathbf{1}_{G,C}(j_c)}} \frac{\phi_I}{\phi_J} \end{aligned} \tag{5}$$

Combining the equations (5) and (4), we get:

$$\begin{aligned} \psi_{\{NI\}} N \rho_{i_c j_c} \pi_J &= \psi_{\{(N-2)I,2J\}} \frac{2(N-2)}{N[(N-2)\gamma^{\mathbf{1}_{G,C}(i_c)}\phi_I + 2\gamma^{\mathbf{1}_{G,C}(j_c)}\phi_J]} \frac{\gamma^{\mathbf{1}_{G,C}(i_c)}}{\gamma^{\mathbf{1}_{G,C}(j_c)}} \frac{\phi_I}{\phi_J} \gamma^{\mathbf{1}_{G,C}(i_c)} \phi_I \\ \Leftrightarrow \psi_{\{NI\}} N \rho_{i_c j_c} \pi_J &= \psi_{\{(N-2)I,2J\}} q_{\{(N-2)I,2J\},\{(N-1)I,J\}} \frac{\gamma^{\mathbf{1}_{G,C}(i_c)}}{\gamma^{\mathbf{1}_{G,C}(j_c)}} \frac{\phi_I}{\phi_J} \\ \Leftrightarrow \psi_{\{NI\}} N \rho_{i_c j_c} \pi_J \frac{\gamma^{\mathbf{1}_{G,C}(j_c)}}{\gamma^{\mathbf{1}_{G,C}(i_c)}} \frac{\phi_J}{\phi_I} &= \psi_{\{(N-2)I,2J\}} q_{\{(N-2)I,2J\},\{(N-1)I,J\}} \end{aligned} \tag{6}$$

Generalising eq. (6) for state $\{(N-n)I, nJ\}$, we get:

$$\psi_{\{NI\}} N \rho_{i_c j_c} \pi_J \frac{\gamma^{\mathbf{1}_{G,C}(j_c)(n-1)}}{\gamma^{\mathbf{1}_{G,C}(i_c)(n-1)}} \frac{\phi_J^{(n-1)}}{\phi_I^{(n-1)}} = \psi_{\{(N-n)I,nJ\}} q_{\{(N-n)I,nJ\},\{(N-n-1)I,(n+1)J\}} \tag{7}$$

Finally, taking it all the way to the last state ($\{NJ\}$), we have:

$$\psi_{\{NI\}} N \rho_{i_c j_c} \pi_J \frac{\gamma^{\mathbf{1}_{G,C}(j_c)(N-1)}}{\gamma^{\mathbf{1}_{G,C}(i_c)(N-1)}} \frac{\phi_J^{(N-1)}}{\phi_I^{(N-1)}} = \psi_{\{NJ\}} q_{\{NJ\},\{I,(N-1)J\}} \Leftrightarrow \frac{\psi_{\{NI\}}}{\psi_{\{NJ\}}} = \frac{\pi_I}{\pi_J} \frac{\gamma^{\mathbf{1}_{G,C}(i_c)(N-1)}}{\gamma^{\mathbf{1}_{G,C}(j_c)(N-1)}} \frac{\phi_I^{N-1}}{\phi_J^{N-1}} \tag{8}$$

To generalise for all monomorphic states, we need to include all codon positions in the GC–bias parameter. Since we are only studying the frequency shifts between codons that differ in a single position, the other two codon positions are the same, therefore have equal GC–biases. In equation (8) the γ parameter can be expanded to:

$$\frac{\gamma^{\mathbf{1}_{G,C}(i_c)(N-1)}}{\gamma^{\mathbf{1}_{G,C}(j_c)(N-1)}} = \frac{\gamma^{\mathbf{1}_{G,C}(i_1)(N-1)}\gamma^{\mathbf{1}_{G,C}(i_2)(N-1)}\gamma^{\mathbf{1}_{G,C}(i_3)(N-1)}}{\gamma^{\mathbf{1}_{G,C}(j_1)(N-1)}\gamma^{\mathbf{1}_{G,C}(j_2)(N-1)}\gamma^{\mathbf{1}_{G,C}(j_3)(N-1)}} = \frac{\gamma^{(N-1)(\mathbf{1}_{G,C}(i_1)+\mathbf{1}_{G,C}(i_2)+\mathbf{1}_{G,C}(i_3))}}{\gamma^{(N-1)(\mathbf{1}_{G,C}(j_1)+\mathbf{1}_{G,C}(j_2)+\mathbf{1}_{G,C}(j_3))}} \tag{9}$$

By incorporating the generalisation for gBGC in equation (9) with the fraction we got in equation (8), we get the approximation for the stationary frequencies for the monomorphic states.

$$\begin{aligned} \psi_{\{NI\}} &\propto \pi_I \gamma^{(N-1)(\mathbf{1}_{G,C}(i_1)+\mathbf{1}_{G,C}(i_2)+\mathbf{1}_{G,C}(i_3))} \phi_I^{N-1} \\ \psi_{\{NJ\}} &\propto \pi_J \gamma^{(N-1)(\mathbf{1}_{G,C}(j_1)+\mathbf{1}_{G,C}(j_2)+\mathbf{1}_{G,C}(j_3))} \phi_J^{N-1} \end{aligned} \tag{10}$$
